# Genome-Wide Transcriptomic Analysis of Non-Tumorigenic Tissues Reveals Aging-Related Prognostic Markers and Drug Targets in Renal Cell Carcinoma

**DOI:** 10.3390/cancers13123045

**Published:** 2021-06-18

**Authors:** Euiyoung Oh, Jun-Hyeong Kim, JungIn Um, Da-Woon Jung, Darren R. Williams, Hyunju Lee

**Affiliations:** 1 School of Electrical Engineering and Computer Science, Gwangju Institute of Science and Technology, 123 Cheomdangwagi-ro, Buk-Gu, Gwangju 61005, Korea; euiyoung.oh@gist.ac.kr; 2 New Drug Targets Laboratory, School of Life Sciences, Gwangju Institute of Science and Technology, 123 Cheomdangwagi-ro, Buk-Gu, Gwangju 61005, Korea; kjhuioph@gist.ac.kr (J.-H.K.); kajamium@gist.ac.kr (J.U.); darren@gist.ac.kr (D.R.W.)

**Keywords:** transcriptomic analysis, normal tissue, aging-related prognostic markers, renal cell carcinoma

## Abstract

**Simple Summary:**

Few studies have employed expression data of normal tissues to explore the survival of cancer patients. Hence, we identified aging-related genes and microRNAs in normal tissues from patients with various types of cancer and investigated their effects on survival. We built statistical models to predict the survival of cancer patients using the expression levels of aging-related genes and microRNAs. We found that five aging-related genes (DUSP22, MAPK14, MAPKAPK3, STAT1, and VCP) from kidney cancer patients were significantly related to patient survival. We experimentally showed that there is an increase in the five aging-related genes in the macrophages of aged mice and, among these five genes, the invasion of cancer cells is inhibited by downregulating DUSP22. Using the zebrafish model of tumor cell dissemination, it was also confirmed that in vivo treatment with a small molecule inhibitor of DUSP22 blocked the metastasis of cancer cells in the early stage.

**Abstract:**

The relationship between expression of aging-related genes in normal tissues and cancer patient survival has not been assessed. We developed a genome-wide transcriptomic analysis approach for normal tissues adjacent to the tumor to identify aging-related transcripts associated with survival outcome, and applied it to 12 cancer types. As a result, five aging-related genes (DUSP22, MAPK14, MAPKAPK3, STAT1, and VCP) in normal tissues were found to be significantly associated with a worse survival outcome in patients with renal cell carcinoma (RCC). This computational approach was investigated using nontumorigenic immune cells purified from young and aged mice. Aged immune cells showed upregulated expression of all five aging-related genes and promoted RCC invasion compared to young immune cells. Further studies revealed DUSP22 as a regulator and druggable target of metastasis. DUSP22 gene knockdown reduced RCC invasion and the small molecule inhibitor BML-260 prevented RCC dissemination in a tumor/immune cell xenograft model. Overall, these results demonstrate that deciphering the relationship between aging-related gene expression in normal tissues and cancer patient survival can provide new prognostic markers, regulators of tumorigenesis and novel targets for drug development.

## 1. Introduction

Cancer is considered an aging-associated degenerative disease. The mechanism by which aging contributes to cancer progression is a topic of active investigation. Numerous cellular events associated with aging, such as genomic instability, DNA damage, inflammation, and immune system impairment, are also known as hallmarks of cancer [1,2,3]. Recently, the contribution of the aging microenvironment to cancer has been extensively studied, providing new insights into cancer progression [4]. Senescence in cells increases the secretion of senescence-associated cytokines, chemokines, and growth factors, which drive tumor cell invasion [5]. The extracellular matrix (ECM) integrity decreases with aging, and changes in the ECM are related to tumor metastasis [6,7].

Although the aging microenvironment has been studied in cancer, few studies have investigated how changes in the expression of aging-related genes in the normal tissues of cancer patients are related to cancer progression on a genome-wide scale. Several previous studies have characterized aging-related genes and their biological functions. For example, Yang et al. analyzed synchronized age-related gene expression changes across multiple tissues and their potential link to degenerative diseases in the Genotype-Tissue Expression (GTEx) project [8,9,10]. While the incidence of most cancer types increases with age, some cancer types, such as breast cancer, are more aggressive in younger patients [11]. However, elderly patients with thyroid or prostate cancer have poor survival rates [12,13]. To enhance our understanding of aging and cancer, it is important to investigate the relationship between the expression levels of aging-related genes in normal tissues and cancer progression or invasion.

In this study, we investigated the relationship between genome-wide expression changes of aging-related genes in the normal tissues of cancer patients and their subsequent survival in 12 cancer types described in The Cancer Genome Atlas (TCGA) [14,15,16,17,18,19,20]. We first developed an approach to identify a group of aging-related genes by integrating gene expression data of normal tissues of cancer patients and protein–protein interaction data. We then examined whether the expression levels of aging-related genes are related to the survival of cancer patients. Furthermore, the effects of identified genes on cancer invasion and metastasis, which are major causes of cancer morbidity and mortality, were initially validated in cell-based models of young and aged immune cell types purified from normal tissues. Further characterization was undertaken in a mixed immune and tumor cell xenograft model that allowed quantification of the early stages of metastasis.

This transcriptomic methodology identified prognostic markers in kidney clear cell carcinoma, the most common form of renal cell carcinoma (RCC). Investigation of gene function in cell-based and xenograft models of RCC progression revealed dual specificity phosphatase 22 (DUSP22) as a novel regulator and drug target for RCC metastasis. RCC patients are often diagnosed with metastatic disease, which is associated with a 5-year survival rate under 10%, and RCC causes 15,000 deaths annually in the United States [21].

As more data regarding cancer patient survival and gene expression in normal tissues becomes available, the novel transcriptomic methodology described herein can facilitate the discovery of prognostic markers, regulators of cancer progression, and therapeutic targets.

## 2. Materials and Methods

### 2.1. Overview of the Proposed Approach

**Figure 1:**
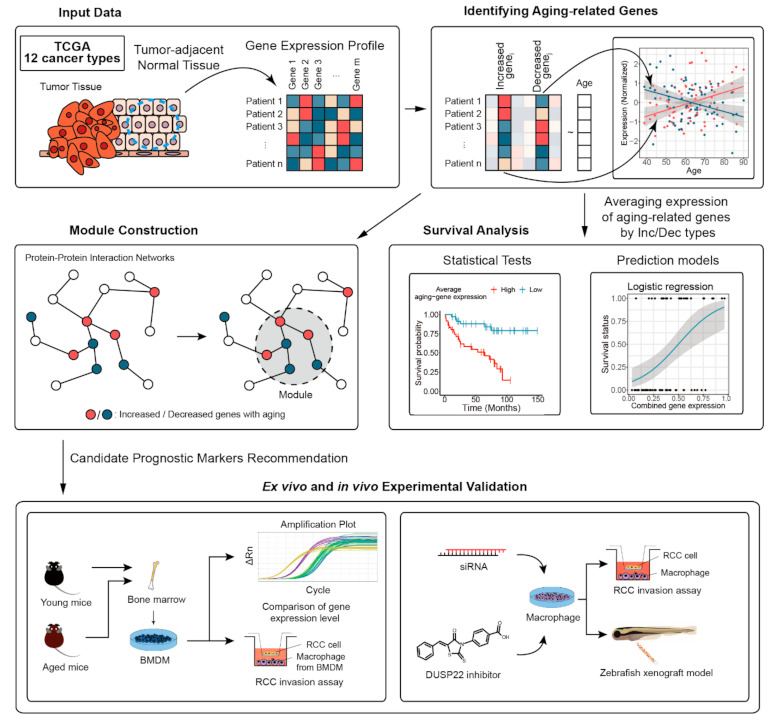
Overall workflow of the study. Aging-related genes were identified from 12 TCGA transcriptomic datasets by performing linear regression. We constructed modules containing aging-related genes to find representative clusters associated with aging. We analyzed relationships between aging-related genes and survival of patients by statistical tests and prediction models. Ex vivo and in vivo experiments using an animal model of aging were carried out to validate aging-related expression and its effect on cancer cell invasion and metastasis.

The overall workflow is depicted in Figure 1. Gene expression datasets of normal and tumor cells of cancer patients were collected from TCGA [14,15,16,17,18,19,20]. Selected cancer types and their abbreviations are as follows: bladder cancer (BLCA), breast cancer (BRCA), kidney-related cancer (KICH, KIRC, KIRP), lung-related cancer (LUAD, LUSC), head and neck cancer (HNSC), liver cancer (LIHC), stomach cancer (STAD), thyroid cancer (THCA), and uterine cancer (UCEC). Aging-related genes and microRNA (miRNA) were identified from comparing normal and tumor transcriptomic datasets using linear regression and DESeq2 (RRID:SCR_000154), respectively [8,22,23,24]. Aging-related genes on protein–protein interaction networks and constructed modules were mapped to find representative clusters associated with aging, by using a dynamic tree cut method [25]. Enriched biological functions and survival prediction performances of the identified aging-related genes were investigated using DAVID (RRID:SCR_001881) and logistic regression models, respectively [26]. The relationship between expression levels of identified genes and patient survival was investigated via Cox regression and the Kaplan–Meier estimator. An animal model of aging was utilized to validate the effect of expression of each identified aging-related gene on cancer cell invasion and metastasis.

### 2.2. Materials

We compared the mRNA and miRNA expression profiles between normal and tumor tissues derived from the same patient suffering a type of cancer among the dataset types BLCA, BRCA, KICH, KIRC, KIRP, LUAD, LUSC, HNSC, LIHC, STAD, THCA, and UCEC. The expression of mRNAs and miRNAs were determined using Illumina HiSeq–RNASeqV2 and Illumina HiSeq–miRNASeq, respectively. We used fragments per kilobase of transcript per million mapped reads upper quartile (FPKM-UQ) for identifying aging-related genes in normal tissues and DEGs in tumor tissues, respectively. The miRNA expression results were recorded in read counts and reads per million reads (RPM). Data were downloaded from the TCGA Data Portal using TCGAbiolinks [27].

### 2.3. Data Pre-Processing

TCGA transcriptomic profiling data contains protein coding genes and noncoding regions like pseudogenes and noncoding RNAs. Based on the Ensembl (RRID:SCR_002344) gene biotype and after filtering out noncoding regions, we obtained 19,589 protein coding genes. Then, we selected genes with nonzero expression values in more than 30 samples to reduce the number of unreliable results in each tissue type. If the number of samples was less than 30, we removed genes in which expression values were zero in more than 30% of the samples. Because miRNA expression values were represented in RPM values, we used quantile normalization for miRNA expression data to allow comparisons between samples. Furthermore, we filtered out miRNAs if more than 70% of the samples had a zero expression value of the miRNA.

### 2.4. Identifying Aging-Related Genes and miRNAs

The following methods for identifying aging-related genes and miRNAs were based on previous studies that utilized linear models [8,22,23]. We used linear regression models to identify aging-related genes and miRNAs because aging is a continuous process, not a discrete event. We employed the two following linear regression models:

(1) $$M1:Expression_{ij}=\beta_{j}+\gamma_{j}Age_{i}+\epsilon_{ij},$$

(2) $$M2:Expression_{ij}=\beta_{j}+\gamma_{j}Age_{i}+\sum_{k=1}^{N}\alpha j_{k}PCi_{k}+\epsilon_{ij},$$

where $Expression_{ij}$ denotes the expression value of the gene or miRNA j in sample i, $Age_{i}$ denotes the age at initial diagnosis of sample i, β is the regression intercept for gene j, $\gamma_{j}$ is the regression coefficient of age, and $\epsilon_{ij}$ is the error term. $PC_{jk}$ denotes the value of the k-th principal component (PC) value of gene expression data for the *i*-th sample. Among the top ten PCs, we selected N PCs that were not significantly correlated with age (*p*-values for Pearson’s correlation test > 0.05) to increase the significance level of the age coefficient. 

We selected genes and miRNAs with an age regression coefficient that corresponded to *p*-values of below 0.05 in both linear regression models. Aging-related genes and miRNAs were classified as increasing or decreasing with age depending on the sign of the age coefficient in linear regression models. To confirm that the detected aging-related genes were not sensitive to a certain configuration, we resampled data using the bootstrapping method. Details about the bootstrapping are described in Appendix A.1. After confirming aging-related genes, the average expression values from increasing/decreasing genes were calculated and referred to as the “increasing or decreasing index”, respectively.

To investigate whether the expression value of identified aging-related genes significantly affected the survival time of cancer patients, Cox proportional hazards models and Kaplan–Meier estimators were used [28,29]. Details about survival analysis steps are described in Appendix A.2.

### 2.5. Finding DEGs in Cancer

We employed DESeq2 (RRID:SCR_000154) to find DEGs in cancer [24]. Expression data from both tumor and normal tissues in the same patient were paired. We constructed DESeq2 models in which gene expression was set as a dependent variable and tissue type and patient ID as independent variables. A gene was considered differentially expressed if a log 2-fold-change between normal and cancer tissues was > 2 and the adjusted *p*-value, determined by a Benjamini–Hochberg method, was < 0.01.

### 2.6. Building Modules Using Protein–Protein Interaction Networks

To build modules including functionally-related aging genes, we used the protein–protein interaction database provided by the HPRD [30]. We compiled a protein–protein interaction database from HPRD, constructed the network, and mapped aging-related genes. To calculate the similarity distance between any two aging-related genes in the network, we used the diffusion kernels method [31]. We constructed a hierarchical cluster tree based on the dissimilarity between aging-related genes, and defined modules using the dynamic tree cut method [25]. Details about diffusion kernels are described in Appendix A.3. 

### 2.7. Experimental Validation

#### 2.7.1. Experimental Reagents

Collagen type I was purchased from Roche (Basel, Switzerland). Lipofectamine 3000 transfection kit (L300008), Silencer negative control siRNA (AM4611), mouse Dusp22 siRNA (#287290), and Mapk14 siRNA (#240556) were obtained from Invitrogen (Waltham, MA, USA). The anti-CD68 (ab201340) and anti-cytokeratin (ab9377) antibodies were purchased from Abcam (Cambridge, UK). BML-260(sc-223822A) was purchased from Santa Cruz Biotechnology (Dallas, TX, USA).

#### 2.7.2. Cell Culture

RENCA murine renal epithelial adenocarcinoma cells and RAW264.7 murine macrophage cells were purchased from the Korean Cell Line Bank (Seoul, Korea). RENCA and RAW264.7 cells were maintained in DMEM media (Gibco, Thermo Fisher Scientific, Waltham, MA, USA), supplemented with 10% FBS and 1% penicillin/streptomycin.

#### 2.7.3. Collection of Conditioned Media

To collect conditioned media (CM), cells were grown to 70~80% confluency in 10 cm culture dishes, and the media was changed to serum-free media. Then 48 h later, the CM was collected and centrifuged at 1500 rpm for 3 min at 4 °C. The CM was then filtered using a 0.2 μm syringe filter and stored at −20 °C until use.

#### 2.7.4. siRNA-Mediated Gene Knockdown

Cells were seeded into 6-well plates for RNA isolation and 24-well plates for the invasion assay. After incubation for 24 h, the transfection media was prepared by using Lipofectamine 3000. Lipofectamine 3000 and siRNA were diluted in DMEM, mixed at a 1:1 ratio, and incubated at room temperature (RT) for 15 min. Cells were then washed with PBS and transfection media was treated with the appropriate volume. Experiments were performed 24 h after transfection.

#### 2.7.5. Real-Time Quantitative PCR

Each cDNA was synthesized from RNA using AccuPower RT PreMix (Bioneer, Daejeon, Korea), according to the manufacturer’s protocol. Each sample was tested in triplicate by qPCR in a total volume of 20 μL containing 10 μL ToPreal qPCR 2× PreMIX (Enzynomics, Daejeon, Korea), 250 nM specific forward and reverse primers, and 1 μL cDNA. The initial denaturation step was performed at 95 °C for 10 min, and the amplification stage comprised 40 cycles of denaturation, annealing, and extension. Denaturation was carried out at 95 °C for 15 s, annealing and extension were performed at 60 °C for 1 min. After the last cycle, the melting points of all samples were analyzed within the range of 60–95 °C through continued fluorescence detection. Gene expression was normalized with that of GAPDH. Details of the primers are shown in in Table S7.

#### 2.7.6. Invasion Assay

The cancer cell invasion assay was performed in 24-well transwell plates (Corning Costar, New York, NY, USA). The 8-μm pore size transwell was coated with type I collagen (3 μg/60 μL/well). A total of 4 × 10^4^ BMDM cells were seeded in the lower chamber and differentiated into macrophages using cancer CM. A total of 1 × 10^4^ cancer cells were seeded in the upper transwell. After 24 or 48 h, cancer cells that invaded the lower chamber through the porous membrane were fixed with 3.7% formaldehyde and stained with 0.2% crystal violet. The images of stained cells were obtained using a light microscope (CKX41, Olympus, Tokyo, Japan) and analyzed by ImageJ software (NIH, Bethesda, MD, RRID:SCR_003070). For assessing the effects of DUSP22 or MAPK14 knockdown, 1 × 10^5^ RAW264.7 cells were seeded in the lower chamber and transfected with siRNA.

#### 2.7.7. Immunocytochemistry

BMDM were differentiated in 24-well culture plates for 3 d using cancer CM. Cells were immunostained for CD68, a pan-macrophage marker, or cytokeratin, an epithelial cell marker [32]. Cells were fixed using 3.7% paraformaldehyde solution in 1× PBS at RT for 10 min. The cells were washed using PBST solution (1× PBS with 0.1% Tween-20) and then permeabilized with 0.25% Triton X-100 in 1× PBS for 10 min at RT. After washing, unspecific binding of the antibodies to cells was blocked with 1% BSA, 22.52 mg/mL glycine in PBST for 30 min at RT and incubated with CD68 or cytokeratin antibody in 1% BSA in PBST overnight at 4 °C. Alexa Fluor 488 goat anti-mouse IgG or Alexa Fluor 594 goat anti-rabbit IgG was used as a secondary antibody (Abcam, Cambridge, UK). Secondary antibody in 1% BSA in PBST was applied to the cells for 1 h at RT. Nuclei were stained using DAPI. Staining was visualized by fluorescence microscopy (Leica DMI3000 B).

#### 2.7.8. The Enzyme-Linked Immunosorbent Assay (ELISA)

ELISA for conditioned media was performed using a commercial kit, according to the manufacturer’s protocol (M-CSF and GM-CSF kits were purchased from R&D systems). To quantify protein levels of M-CSF or GM-CSF, conditioned media were harvested after 48 h of culture in serum-free media.

#### 2.7.9. Animals

Animal experiments were approved by the Animal Care and Use Committee of the Gwangju Institute of Science and Technology (GIST-2019-042). Mice were supplied by Damool Science (Daejeon, Republic of Korea) and Orient Bio, Inc. (Gyeonggi, Republic of Korea) To perform in vitro experiments, BMDM were isolated from the femur and tibia bones of C57BL/6 male mice. The bone marrow cells were collected from these bones using a 30 G syringe and ice-cold sterile PBS. The cells were then filtered through a 70-μm cell strainer to remove tissue debris and centrifuged at 300× *g* for 10 min at 4 °C. Red blood cells were eliminated from the pellet by adding 5 mL red blood cell lysis buffer for 5 min, and centrifuging the sample after adding 5 mL PBS. BMDM cells were maintained in 6-well ultra-low attachment plates with monocyte medium (RPMI1640 supplemented with 10% fetal bovine serum, 1% penicillin/streptomycin, 1× Glutamax (Gibco, Thermo Fisher Scientific), 1 mM sodium pyruvate, 1× non-essential amino acids, 10 mM HEPES, 55 μM β-mercaptoethanol, and 10 ng/mL M-CSF (Peprotech, Rocky Hill, NJ, USA)) at 37 °C for 5 d. Monocytes were purified by negative selection with anti-CD117 magnetic microbeads (Miltenyi Biotec, Bergisch Gladbach, Germany) using a magnet separator to remove non-monocytes from the cell population. 

#### 2.7.10. Zebrafish–Human Cancer Xenograft Model

Assessment of mouse RCC cell dissemination (an early stage of metastasis) in vivo was carried out using a validated zebrafish model [33]. Embryos were staged for cell xenoplantation at 48 h postfertilization and 39 or 44 embryos were used for each treatment group. After RCC cells were stained with 2 μg/mL DiI (Invitrogen), the embryos were dechorionized and anesthetized with 0.0016% tricaine. Two hundred RCC cells were injected, or RCC cell and DMSO-treated macrophages, or a RCC cell and DUSP inhibitor-treated macrophage cell mix (1:1 ratio) were injected into the center of the yolk sac using an injector (Picospritzer III, Parker Hannifin, Cleveland, OH, USA). Xenografted embryos were transferred to a 96-well plate in 200 μL E3 medium. The number of embryos exhibiting dissemination from the injection site was counted using fluorescence microscopy (Leica DM2500 Microscope).

#### 2.7.11. Statistics for Cell-Based Analysis

For cell-based analyses, statistical analysis was performed using Excel (Microsoft 2016). The parametric Student’s t-test was used for statistical analysis. A *p*-value of < 0.05 was considered significant. 

## 3. Results

### 3.1. Characteristics of the Data

#### 3.1.1. Demographics

The number of samples, the demographic characteristics and survival rates of patients with specific cancer types are shown in Table 1. Patients’ ages ranged from 20 to 90 years, with 60 years as the most frequent. The mean and the standard deviation for age was 61 ± 14.8.

#### 3.1.2. Survival Analysis with Chronological Age

To investigate whether patient age and survival were related, we built a univariate Cox regression model to predict survival by using age as an input variable for each cancer type. The results showed that age was a significant risk factor in KIRC and THCA, with *p*-values of 0.002 and 0.026, respectively. In contrast, age was not related to survival in other cancer types, as *p*-values were not significant according to the univariate Cox regression model (Table S1).

### 3.2. Tissue-Specific Aging-Related Genes and miRNAs

The number of identified aging-related genes and miRNAs per cancer type is shown in Table 1 and the detailed list is described in Tables S2 and S3. Among the aging-related genes and miRNAs, the expression of only 20 genes (12 upregulated and 8 downregulated) and six miRNAs (2 upregulated and 4 downregulated) were identified as significantly different in more than three tissue types, implying that the alteration in expression levels during aging was highly tissue-specific. Especially, ZNF518B was identified in six tissue types, BRCA, KICH, LIHC, LUSC, KIRP, and THCA, and NEFH was detected in four tissue types, BRCA, KIRC, LUAD, and THCA. 

**Figure A1:**
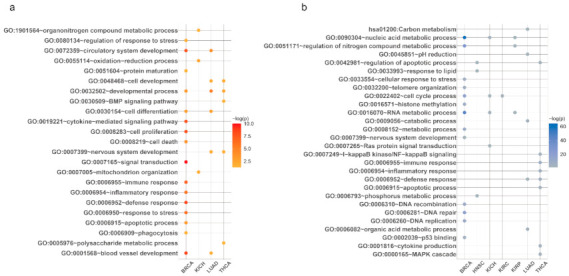
Examples of aging-related pathways. Representative pathways which were enriched by (**a**) increasing and (**b**) decreasing aging-related genes identified from each tissue type.

Pathway enrichment tests were performed for aging-related genes by each tissue and increasing/decreasing types. We utilized Database for Annotation, Visualization and Integrated Discovery (DAVID) v6.7 tools for Gene Ontology (GO), and the Kyoto Encyclopedia of Genes and Genomes (KEGG) [26,34,35,36]. Notably, immune system, cell cycle, and metabolic process-related pathways were frequently enriched with aging-related genes. These biological processes are also known to be related to cancer occurrences [37], showing a relationship between the expression of aging genes and cancer development. However, in general, the results showed that the altered aging-related pathways were different in each tissue type. Representative aging and cancer-related pathways are depicted in Figure A1, and all annotated pathways for aging-related genes are shown in Table S4.

### 3.3. Association between Survival and Aging-Related Genes in BLCA, BRCA, and THCA

We investigated the relationship between aging-related genes and patient survival by each tissue and increasing/decreasing types. For BLCA, BRCA, THCA, expression levels of decreasing aging-related genes were significantly related with patient survival. Survival analysis results for BLCA, BRCA, and THCA are described in Appendix A.4, and for all cancer types, are shown in Table S5.

### 3.4. Analysis of KIRC 

#### 3.4.1. Association between Survival and Age-Related Genes in TCGA-KIRC

In KIRC, the average expression of all aging-related transcripts was related to survival. The increasing index, which derived from 162 upregulated genes, had a hazard ratio of 2.27 and a *p*-value of 2.09 × 10^−5^, whereas the decreasing index, derived from 87 downregulated genes, had a hazard ratio of 0.48 and *p*-value of 0.01 in the univariate Cox models.

**Figure 2:**
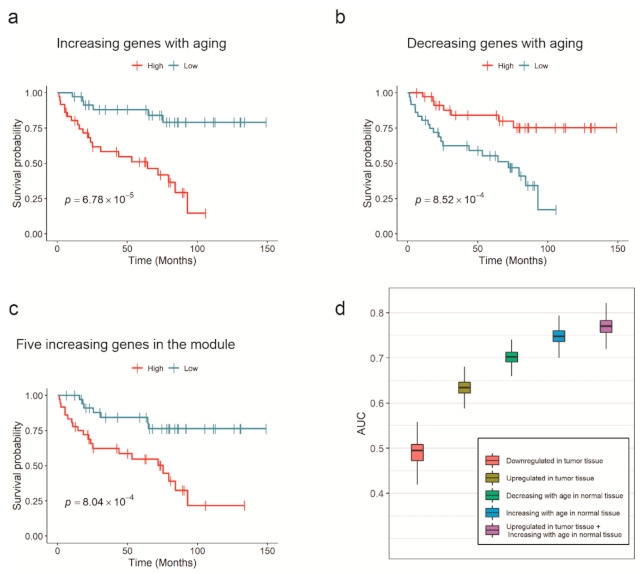
Survival analysis and prognostic capability of aging-related genes in KIRC. (**a**–**d**) Kaplan–Meier overall survival curve of aging-related genes or miRNAs. The patients were grouped by the average expression of: (**a**) increasing genes and (**b**) decreasing genes in TCGA-KIRC. (**c**) The average expression value of five upregulated genes in the module (*DUSP22*, *MAPK14*, *MAPKAPK3*, *STAT1*, and *VCP*) also had a predictive power. (**d**) AUCs from survival prediction models with five different input variables were compared. Expression levels of aging-related genes in normal kidney tissue exhibited better survival prediction performance than DEGs in KIRC tissue. The best performance was achieved when both aging-related genes and DEGs were simultaneously used.

When the patients were divided into two groups based on the median of the increasing or decreasing indices, the Kaplan–Meier estimator results agreed with those of the Cox models. The Kaplan–Meier model exhibited a *p*-value of 6.78 × 10^−5^ or 8.52 × 10^−4^ in the log-rank test when the criterion was the increasing or decreasing index, respectively. The Kaplan–Meier overall survival curve of aging-related genes in TCGA-KIRC patients is plotted in Figure 2a,b. These results demonstrated that a kidney cancer patient with a younger gene expression pattern in normal cells has a higher probability of living longer.

Among the identified aging-related miRNAs in KIRC, 17 were upregulated and 15 downregulated. The increasing index from 17 upregulated miRNAs had a hazard ratio of 1.67 and a *p*-value of 0.008, and the decreasing index from the 15 downregulated miRNAs had a hazard ratio of 0.44 and a *p*-value of 7.89 × 10^−4^ in the Cox model. The *p*-value of the Kaplan–Meier model, when increasing and decreasing indices were used as independent variables, were 0.005 and 0.03, respectively. These results were consistent with those showing that age was a significant risk factor in KIRC. Notably, KIRC miRNAs, similarly to aging-related genes, were associated with survival. Based on these results, we focused on KIRC for further analysis.

#### 3.4.2. Module Analysis 

To find representative clusters of aging-related genes, a protein–protein interaction network was constructed including the 73 upregulated and 29 downregulated aging genes from KIRC, by using the Human Protein Reference Database (HPRD). Based on the dissimilarities between the mapped aging-related genes, five upregulated (DUSP22, MAPK14, MAPKAPK3, STAT1, and VCP) and three downregulated (BRCA1, BRIP1, and NUFIP1) genes were detected. These genes functioned as DNA damage checkpoints (Benjamini adjusted *p*-value of 0.02), intracellular signaling mediators (0.02), and cell cycle checkpoints (0.03). Alterations of these biological pathways were observed in renal carcinoma patients [38,39].

Interestingly, the average expression of the five upregulated genes in KIRC was also related to survival. The increasing index derived from DUSP22, MAPK14, MAPKAPK3, STAT1, and VCP had a hazard ratio of 1.86 and a *p*-value of 5.37 × 10^−5^ in the Cox model. At the same time, the Kaplan–Meier estimator showed a *p*-value of 8.04 × 10^−4^, as presented in Figure 2c. In fact, the expression value of each of DUSP22, MAPKAPK3, VCP, and STAT1 had a significant relationship with survival (with a Cox-regression *p*-value of 0.01, 0.04, 0.02, and 5 × 10^−4^, respectively), while MAPK14 did not.

#### 3.4.3. Validation of Survival Significance in Kidney Renal Cells with an Independent Dataset

To validate the survival significance of aging-related genes in kidney renal cells, analysis with an independent dataset named the Renal Cell Cancer–EU/FR (RECA-EU) from the International Cancer Genome Consortium was performed. RECA-EU provided gene expression data of normal kidney renal cells from 45 RCC patients, of whom 17 were deceased. The mean age of the patients was 61, with a standard deviation of 10. In contrast to TCGA-KIRC, the survival status of subjects in the RECA-EU dataset was not related to age (*p*-value of 0.57 in the univariate Cox model). Among 162 upregulated and 87 downregulated aging genes in TCGA-KIRC, RECA-EU had probes corresponding to 160 upregulated and 85 downregulated genes. Although aging-related genes from TCGA-KIRC were not correlated with age in RECA-EU, the results of the univariate Cox model showed that the increasing index in RECA-EU also had prognostic power of survival, with a hazard ratio of 1.60 and a *p*-value 0.036, which was consistent with the TCGA-KIRC results. 

**Figure 3:**
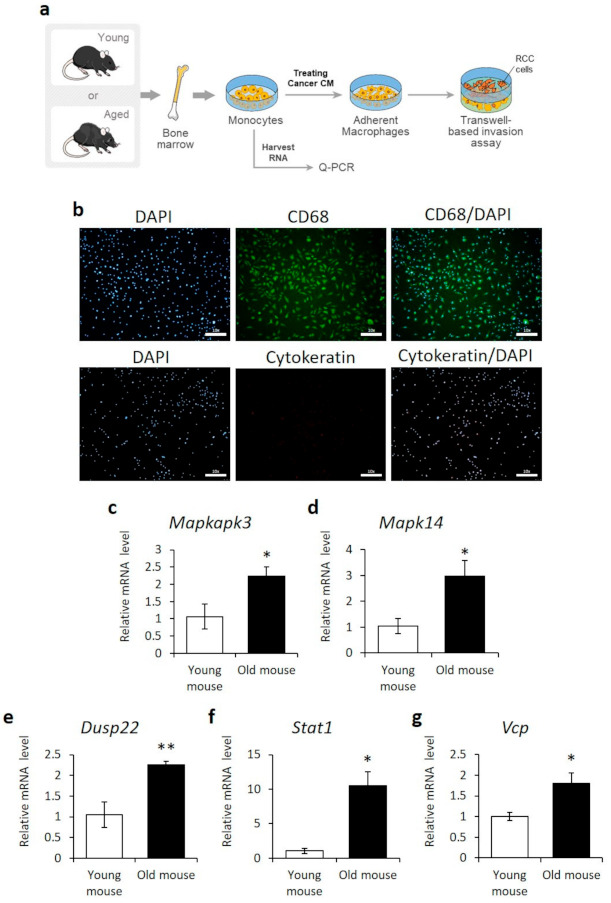
Upregulation of *MAPKAPK3, MAPK14, DUSP22, STAT1,* and *VCP* in the bone marrow-derived monocytes/macrophages of old mice. (**a**) Schematic diagram of the protocol assessing the aging-related genes in monocyte/macrophage derived from young or aged mice. (**b**) Immunofluorescence staining of CD68 and cytokeratin in primary bone marrow-derived macrophages differentiated by RENCA cell conditioned media. Nuclei were visualized using DAPI. Scale bar = 100 μm. (**c**–**g**) qPCR analysis of *MAPKAPK3*, *MAPK14*, *DUSP22*, *STAT1*, and *VCP* gene expression in primary bone marrow-derived monocytes isolated from young (5-week-old) or old (72-week-old) mice. *n* = 3, Error = SE, * = *p*-value < 0.05, ** = *p*-value < 0.01 compared to young mice.

The Kaplan–Meier model showed a *p*-value of 0.04 in the log-rank test when the criterion was the increasing index. The survival curve is plotted in Figure 3d. However, downregulated aging genes identified from TCGA-KIRC did not have prognostic power in RECA-EU (*p*-value of 0.08, according to the Cox model).

Lastly, a Cox regression test was performed using the five upregulated aging genes identified from the TCGA-KIRC data (DUSP22, MAPK14, MAPKAPK3, STAT1, and VCP), in the RECA-EU dataset and determined a significant relationship between their expression and patient survival (*p*-value of 0.02 and a hazard ratio of 1.7).

#### 3.4.4. Biological Roles of Aging-Related miRNAs in the Kidney

A literature search of the biological roles of aging-related miRNAs was performed. Bai et al. observed that overexpression of miR-335 and miR-34a induced premature senescence of young mesangial cells in the kidney via suppression of the mitochondrial antioxidative enzymes SOD2 and TXNRD2, with a concomitant increase in reactive oxygen species levels [40]. Chen et al. demonstrated that downregulation of miR-136-5p promoted cell proliferation, migration, and invasion, whereas it suppressed cell apoptosis in RCC [41]. 

#### 3.4.5. Deferentially Expressed Genes (DEGs) in Kidney Cancer

A total of 423 upregulated and 932 downregulated DEGs were identified in 72 KIRC patients by using DESeq2 tools (RRID:SCR_000154). Upregulated genes were enriched in immune system and cytokine-related pathways, as shown in Table S6, whereas downregulated genes had no significantly enriched pathways. 

A total of 19 aging-related genes were kidney cancer DEGs: ABCG8, ADGRV1, CDH3, CGA, CPAMD8, CRISP2, DNER, ERP27, GABRP, LHFPL4, OR2I1P, PAPPA2, PCSK9, S100A2, SCEL, SLC16A5, STAP1, TMPRSS4, and UBD. In addition, the average expression levels of 423 upregulated DEGs in tumor tissue were correlated with survival in the Cox model, with a hazard ratio of 1.69 and a *p*-value of 0.01. However, the average expression of 932 downregulated DEGs in tumor tissues corresponded to a hazard ratio of 0.87 and thus indicated the absence of a significant relationship with survival.

#### 3.4.6. Survival Prediction Models

To compare the prognostic power of aging-related genes and cancer DEGs in KIRC, survival, prediction models were developed using logistic regression and five input variables:

1. The average expression of downregulated cancer DEGs in tumor tissue;
2. The average expression of upregulated cancer DEGs in tumor tissue;
3. Decreasing index in normal tissue;
4. Increasing index in normal tissue;
5. The combination of 2 and 4.

Survival status was used as a dependent variable in all cases. We performed a five-fold cross-validation 1000 times for each model, because the number of samples in KIRC (N = 72) was not sufficient to produce consistent performance scores, owing to randomness when dividing these data between the training and test sets. The average area under the ROC curves (AUCs) of models that used single input variable types from 1 to 4, were 0.483, 0.633, 0.703, and 0.748, respectively, implying that the expression values of aging-related genes were better survival predictors than cancer DEGs. The increasing index in normal tissue had the best prediction performance; meanwhile, the average expression of downregulated cancer DEGs had the worst performance owing to the lack of a significant relationship with survival, according to the Cox model. In addition, the prediction model built with the combined average expression of upregulated cancer DEGs in tumor tissue and the increasing index in normal tissue as input variables, showed an average AUC of 0.770, which was higher than any single input variable model. These results suggested that combined information from both cancer and normal tissues was more effective for predicting survival than using them separately. Boxplots of the calculated AUCs are shown in Figure 2d.

To avoid decreasing the significance level of correlated variables, known as multicollinearity, increasing and decreasing indices were not used simultaneously due to their significant correlation.

### 3.5. Experimental Validation

#### 3.5.1. Upregulated Expression of DUSP22, MAPK14, MAPKAPK3, STAT1, and VCP Genes in Aged Mouse Primary Bone Marrow-Derived Macrophages (BMDM)

To validate the transcriptome analysis, PCR-based gene expression analysis was carried out in BMDM isolated from young (5 weeks) and old (72 weeks) mice. BMDM were analyzed because: (1) they are derived from normal tissue; (2) they contribute to the immune response as tumor-associated macrophages (TAMs), which are abundantly present at tumor sites; and (3) they are commonly used for gene biomarker analysis in humans [42,43]. BMDM from young and old mice were differentiated into macrophages by culture with cancer conditioned media (Figure 3a). Macrophage differentiation was confirmed by immunostaining for the marker CD68 (Figure 3b) and qPCR for F4/80, CD68, CD206, and iNOS (Figure S1). The secretion of macrophage differentiating factors by kidney carcinoma cells was confirmed using ELISA (Figure S2). The expression levels of the DUSP22, MAPK14, MAPKAPK3, STAT1, and VCP genes were compared by qPCR. All aging-related genes identified by the transcriptome analysis showed more than a two-fold increase in expression in macrophages derived from old BMDM, compared to that in young BMDM (Figure 3c–g).

#### 3.5.2. Inhibited RCC Invasion by DUSP22 Knockdown in Macrophages Derived from Old Mice 

**Figure 4:**
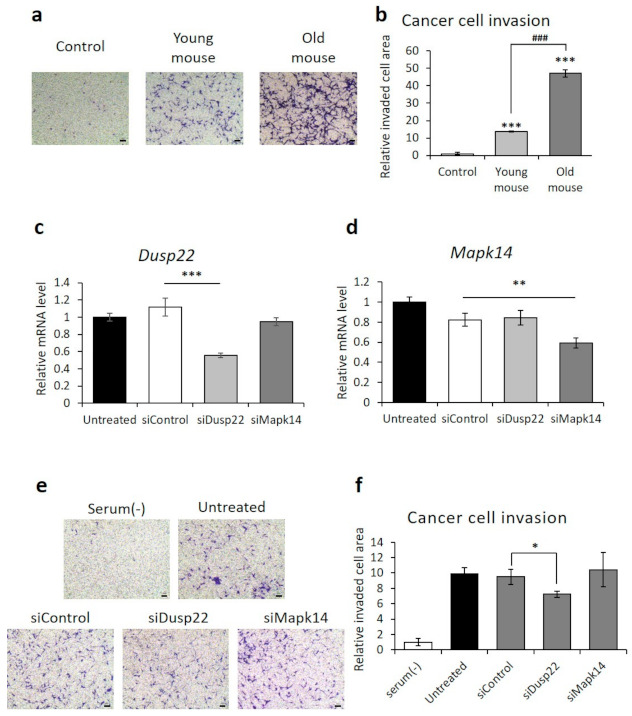
Reduced cancer cell invasion after knockdown of *DUSP22*. (**a**,**b**) Representative images and quantification of invaded RENCA renal adenocarcinoma cells cocultured with primary bone marrow-derived macrophages isolated from young or old mice. Scale bar = 100 μm. (**c**,**d**) qPCR analysis of *DUSP22* and *MAPK14* expression in RAW264.7 cells after transfection with a scrambled control, *DUSP22*, or *MAPK14* siRNA. (**e**,**f**) Representative images and quantification of invaded RENCA renal adenocarcinoma cells cocultured with RAW264.7 cells transfected with a scrambled control, *DUSP22*, or *MAPK14* siRNA. *n* = 3, Error = SD, *** = *p* < 0.001 compared to untreated control, and ### = *p*-value < 0.001 compared to young mice in (**b**). *n* = 3, Error = SD, *** = *p*-value < 0.001, ** = *p*-value < 0.01, * = *p*-value < 0.05 compared to siControl in (**c**,**d**,**f**). Scale bar = 100 μm.

The survival rate of KIRC cancer patients correlated with the degree of metastasis in TCGA-KIRC, as determined by Fisher’s exact test (*p*-value of 4.4 × 10^−5^). Therefore, we performed cancer cell invasion assays with RCC cells to determine the effect of aging on metastasis. BMDM from old mice induced a more than three-fold increase in RCC invasion, compared to BMDM from young mice (Figure 4a).

Among the five aging-related genes discovered in our TCGA-KIRC analysis, the expression level of DUSP22 was significantly related to metastasis. When the expression levels of DUSP22 were analyzed with a t-test for two groups of KIRC cancer patients with/without metastasis, the *p*-value was 0.04. This result could be explained based on the observation that DUSP22 induced the activation of c-Jun N-terminal kinase (JNK) through the apoptosis signal-regulating kinase 1-MAPK kinase 7-JNK1/2 axis [44]. Activated JNK increased the secretion of epidermal growth factor (EGF) or stromal cell-derived factor 1 (SDF-1/CXCL12), thereby increasing invasion of tumor cells and metastasis [45]. To assess the effect of DUSP22 on RCC cell invasion, DUSP22 knockdown was performed by siRNA. RAW264.7, a murine macrophage cell line, was used instead of BMDM due to the cytotoxicity produced by the transfection reagents. Knockdown of MAPK14, selected as a negative control, did not affect invasion. In contrast, DUSP22 knockdown significantly inhibited RCC cell invasion (Figure 4b,c).

#### 3.5.3. DUSP22 Promotes Macrophage-Induced RCC Metastasis In Vivo

**Figure 5:**
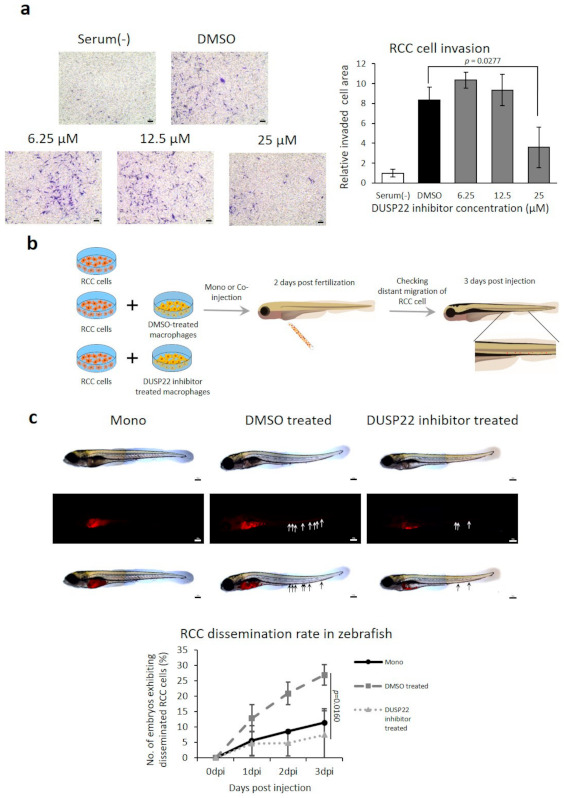
Reduced cancer cell invasion and metastasis in vivo by inhibition of *DUSP22*. (**a**) Inhibitory effect of *DUSP22* targeting drug BML-260 on the invasion assay of RECNA RCC cells cocultured with RAW264.7 macrophages. Scale bar = 100 μm. (**b**) Schematic of the protocol to investigate the effect of macrophage *DUSP22* inhibition on dissemination (early stage of metastasis) in cotransplanted RCC in vivo. (**c**) Representative microscopic images of fish larvae post-transplantation and the RCC dissemination rate with RCC alone (*n* = 39) or cotransplanted with DMSO-treated macrophages (*n* = 39) or BML-260-treated macrophages (*n* = 44). Disseminated RCC cells were visualized by labelling with DiI. Arrows indicate tumor foci disseminated from the injection site (yolk sac). Scale bar = 100 μm.

BML-260 is a small molecule inhibitor of DUSP22 [46]. RCC invasion induced by coculture with macrophages was suppressed by BML-260 (Figure 5a). To investigate the role of DUSP22 in RCC metastasis in vivo, the zebrafish cancer xenograft model was employed (Figure 5b) [33]. Cotransplantation of RCC cells with macrophages produced higher rates of dissemination (the early stage of metastasis) compared to cancer cells alone. Treatment of macrophages with BML-260 prior to cotransplantation with cancer cells significantly inhibited the dissemination rate in vivo (Figure 5c). 

## 4. Discussion

Population aging has increased both the rate of cancer and its impact on the healthcare costs for society. Thus, there is a continued need to identify novel markers, regulators of disease progression and new drug targets for various types of cancer. In this study, we presented a novel transcriptomic methodology to identify gene clusters with expression levels that correlated with aging in the normal tissue adjacent to the tumor, that were also correlated with patient survival. Protein–protein interaction networks including these aging-related genes were constructed to find functionally-related gene clusters. This resulted in the identification of a number of modules for various cancer types. Besides the one module from KIRC that was mentioned in the Results section, seven, one, five, and four modules were found from BRCA, KICH, LUAD, and THCA, respectively. We set the minimum module size to five. For BRCA, the minimum size was set to 25, because the number of identified aging-related genes was greater than that of other tissue types. However, unlike KIRC, no modules from BRCA, KICH, LUAD, and THCA were related to patient survival. Since only four deceased patients were included in each dataset for KICH and THCA, difficulties in performing the survival analysis arose. In the future, we plan to repeat the survival analysis when larger deceased patient numbers become available for KICH and THCA. Thus, the transcriptomic methodology presented in this study has the potential to be further developed when patient survival data in the TCGA becomes available in the future.

In RCC, several mutated genes associated to metabolic, immune, genomic and treatment related external pressure were revealed as principal genes involved in the development and progression of tumors [47], and expression levels of 16 genes were suggested and validated as a recurrence predictor of RCC [48,49]. However, there was no study revealing aging-related genes as prognostic markers of RCC. In this study, among the aging-related genetic predictors of survival in KIRC, we identified DUSP22 as a metastasis-related gene that promoted RCC cell invasion. To our knowledge, these results are the first demonstration that DUSP22 is a prognostic marker and regulator of RCC progression. The mechanism by which DUSP22 can influence RCC invasion can be inferred from previous publications, which showed that DUSP22 activated c-JNK via the apoptosis signal-regulating kinase 1-MAPK kinase 7-JNK1/2 axis. This increases the secretion of two enhancers of cancer cell migration: EGF and stromal cell-derived factor 1 (SDF-1/CXCL12) [44,45]. DUSP22 gene knockdown inhibited the invasion of RENCA cells, which are used as a model of RCC [50]. Therefore, DUSP22 may regulate cancer invasion in other forms of RCC, such as papillary renal cell carcinoma and chromophobe renal cell carcinoma.

The transcriptome analysis was performed in samples obtained from genetically normal tissues that reside next to the tumor and the prognostic markers of survival were validated in BMDM. We selected this cell model because BMDM infiltrate tumor tissue, differentiate into TAMs that reside next to the tumor cells, and are major regulators of cancer progression [51]. Additionally, BMDM biopsies can be readily obtained from RCC patients for genetic analysis. A previous published study investigated the age-related variations in gene expression patterns of RCC [52]. However, the study did not include transcriptomic analysis of patient survival and only reported aging-associated pathways in RCC. 

Although several new therapeutics have been approved in the previous decade, there remains an urgent need to develop new drug targets and increase the therapeutic armamentarium for RCC [53]. There are no reported specific molecular-targeted drugs for RCC [54]. To our knowledge, relatively few DUSP22 inhibitors have been reported in the literature: two rhodamine derivatives, BML-260 and PRL-3 inhibitor I, and the multi-phosphatase inhibitor, PTP inhibitor XIX [55,56]. The results of the current study indicate that DUSP22 may be an attractive target for screening protocols to develop specific drugs for RCC.

RCC is the deadliest type of urogenital tumor [57]. Early diagnosis and prompt treatment would significantly improve patient survival. Moreover, early diagnosis can improve disease progression and help avoid inadequate treatment [57]. Sensitive prognostic biomarkers can facilitate early detection and monitoring of progression [57]. Previous studies have reported prognostic markers for RCC, such as B7-H1, carbonic anhydrase-IX and PTEN (reviewed in [58]). More recent studies have focused on cell-based features, such as aberrant alternative splicing signatures and DNA methylation markers [59,60]. In this study, a novel approach was investigated to discover prognostic markers, based on aging-related genes expressed in normal tissues that are linked to patient survival. The five discovered genes (DUSP22, MAPK14, MAPKAPK3, STAT1, and VCP) have the potential to further improve the current biomarkers developed for RCC (reviewed in [61]), and can be analyzed using biopsies obtained from normal, readily assessed tissues, such as bone marrow. Additionally, these markers may be subjected to follow-up investigation to further characterize the role of immune cells and aging in RCC progression.

## 5. Conclusions

The transcriptomic analysis presented in this study revealed for the first time that aging-related gene expression in normal tissues can predict cancer patient survival. We identified five prognostic markers for RCC that are also expressed in BMDM (DUSP22, MAPK14, MAPKAPK3, STAT1, and VCP) and DUSP22 as a regulator of RCC metastasis and novel target for drug development. These marker genes may improve upon the current set of available biomarkers for RCC. Due to the significant proportion of RCC patients diagnosed with metastatic disease, these results can potentially facilitate kidney cancer diagnosis and therapy. DUSP22 may be an attractive candidate for further development as a specific molecular drug target for RCC. Moreover, this novel approach to transcriptomics can be utilized to identify additional sets of prognostic markers for different cancer types as future patient survival data become available.

## Figures and Tables

**Table 1:** Demographics and the number of identified aging-related genes and microRNAs.

Cancer Type	Sample Size (Survival/Deceased)		Mean Age	Aging Genes			Aging microRNAs		
	Gene	microRNA		Increasing	Decreasing	Total	Increasing	Decreasing	Total
BLCA	19 (8/11)	19 (8/11)	70.32	43	94	137	2	3	5
BRCA	113 (69/44)	104 (61/43)	57.98	772	1678	2450	45	42	87
HNSC	44 (11/33)	44 (11/33)	62.63	52	62	114	5	9	14
KICH	24 (20/4)	25 (21/4)	54.55	202	139	341	5	10	15
KIRC	72 (45/27)	71 (45/26)	62.96	162	87	249	17	15	32
KIRP	32 (25/7)	34 (26/8)	62.40	137	215	352	9	20	29
LIHC	50 (16/34)	50 (16/34)	61.53	13	65	78	8	18	26
LUAD	59 (33/26)	46 (33/13)	65.83	339	289	628	12	11	23
LUSC	49 (19/30)	45 (22/23)	69.25	21	36	57	8	13	21
STAD	32 (23/9)	45 (33/12)	69.25	14	0	14	4	3	7
THCA	58 (54/4)	59 (55/4)	46.03	386	205	591	34	36	70
UCEC	35 (20/3, 12 Not Available)	33 (19/3, 11 Not Available)	59.87	15	6	21	12	16	28

## Data Availability

The datasets analyzed in the study are publicly available in https://portal.gdc.cancer.gov/ (accessed on 17 August 2018). The source codes used for identifying aging-related genes are available in https://github.com/dmcb-gist/Aging_KIRC (accessed on 1 June 2021).

## Supplementary Materials

The following are available online at https://www.mdpi.com/article/10.3390/cancers13123045/s1, Table S1: Summary of Cox regression with age, Table S2: The list of identified aging-related genes in 12 tissue types, Table S3: The list of identified aging-related miRNAs in 12 tissue types, Table S4: DAVID functional annotation of aging-related genes, Table S5: Summary of Cox regression with aging-related genes and miRNAs, Table S6: DAVID functional annotation of upregulated cancer DEGs in KIRC, Table S7: qPCR primers used in this study. Figure S1: qPCR analysis of the pan-macrophage marker (F4/80, CD68), M2 macrophage marker (CD206) and M1 macrophage marker (iNOS) in BMDM isolated from young or old mice, and macrophages differentiated by RENCA cell conditioned media. Figure S2: Quantification of factors associated with macrophage differentiation (M-CSF, GM-CSF) by ELISA in conditioned media from kidney epithelial cells and RENCA cells.

## Appendix A

### Appendix A.1. Bootstrapping

To confirm that the detected aging-related genes were not sensitive to a certain configuration, we used a bootstrapping method. We randomly selected 70% of the samples with replacement and applied the aforementioned linear models 100 times. A gene was determined as an aging-related gene if it satisfied the following conditions:

1. It is an aging-related gene in more than 50 bootstrap runs; and
2. It is an aging-related gene in all samples

However, aging-related miRNAs were not bootstrapped because a very low number of miRNAs remained after bootstrapping.

### Appendix A.2. Survival Analysis

We used a Cox proportional hazards model and a Kaplan–Meier estimator [28,29] to investigate whether the expression value of aging-related genes significantly affected the survival time of cancer patients. Because the number of aging-related genes was larger than the number of samples in most cases, it was difficult to build a survival prediction model if these genes were used as separate input variables. To reduce the amount of expression data, the average gene expression value of the aging-related genes was calculated for each sample. Before calculating the average value, a min–max scaling method was applied for each aging-related gene to reduce the dominant effect of “extremely expressed” genes, with values that were too high or low. After the min–max scaling, the average expression of aging-related genes for each sample was calculated and then Z-normalized. Finally, the average expression values from increasing/decreasing genes were calculated and referred to as the “increasing or decreasing index”, respectively. For miRNAs, increasing and decreasing indices were also calculated. In the Cox regression models, increasing or decreasing indices were used as input variables. In the Kaplan–Meier models, patients were divided into two groups based on the median of the increasing or decreasing indices.

### Appendix A.3. Diffusion Kernel

Diffusion kernel K is defined as follows [31]:

$$K=e^{\left\{\tau H\right\},}$$

where

$$H\left(i,j\right)=\left\{\begin{array}{cc} 1 & \mathrm{if}\;\mathrm{protein}\;i\;\mathrm{interacts}\;\mathrm{with}\;\mathrm{protein}\;j\\ -d_{i} & \mathrm{if}\;\mathrm{protein}\;i\;\mathrm{is}\;\mathrm{the}\;\mathrm{same}\;\mathrm{as}\;\mathrm{protein}\;j\\ 0 & \mathrm{otherwise}, \end{array}\right.$$

where $d_{i}$ is the number of interaction partners for protein i, τ is the diffusion constant, and $e^{\left\{H\right\}}$ represents the matrix exponential of the adjacent matrix H. Dissimilarity is defined as

Dissimilarity=1−K

where K indicates the similarity between genes.

### Appendix A.4. Association between Survival and Downregulation of Age-Related Genes in BLCA, BRCA, and THCA

For convenience, the average gene expression values derived from increasing/decreasing protein genes were referred to as the “increasing/decreasing index”, respectively. After investigating the relationship between aging-related genes and miRNAs from 12 tissues and survival, we determined that decreased expression of genes and miRNAs was significantly correlated to survival in BLCA, BRCA, THCA, and KIRC.

In BLCA, the average expression of downregulated genes was significantly related to survival. According to the univariate Cox models, the decreasing index, which derived from the analysis of 94 downregulated genes, had a hazard ratio of 0.41 and a *p*-value of 0.04. In addition, the Kaplan–Meier model showed a *p*-value of 0.03. On the other hand, in BRCA and THCA, the average expression of downregulated miRNAs was significantly related to survival. The decreasing index from 42 downregulated miRNAs in BRCA and 36 downregulated miRNAs in THCA had hazard ratios of 0.65 and 0.065 and *p*-values of 0.034 and 0.027, respectively. Especially, the Kaplan–Meier model of the decreasing index in BRCA had a *p*-value of 1.5 × 10^−3^. Detailed results of the Cox regression models of other tissues are shown in Table S5.
