# Supplementary material for: Genome-Wide Transcriptomic Analysis of Non-Tumorigenic Tissues Reveals Aging-Related Prognostic Markers and Drug Targets in Renal Cell Carcinoma
Source: Cancers (Basel). 2021 Jun 18;13(12):3045. doi: 10.3390/cancers13123045 (PMC8234889; doi:10.3390/cancers13123045)
Supplement: Supplementary file 1 [file cancers-13-03045-s001.zip › Supplementary Figures.pdf]

Supplementary Figures

Genome-wide transcriptomic analysis of non-tumorigenic tissues reveals aging-related prognostic markers and drug targets in renal cell carcinoma

Euiyoung Oh[1#], Jun-Hyeong Kim[2#], JungIn Um[2], Da-Woon Jung[2*], Darren R. Williams[2*], and Hyunju Lee[1*]

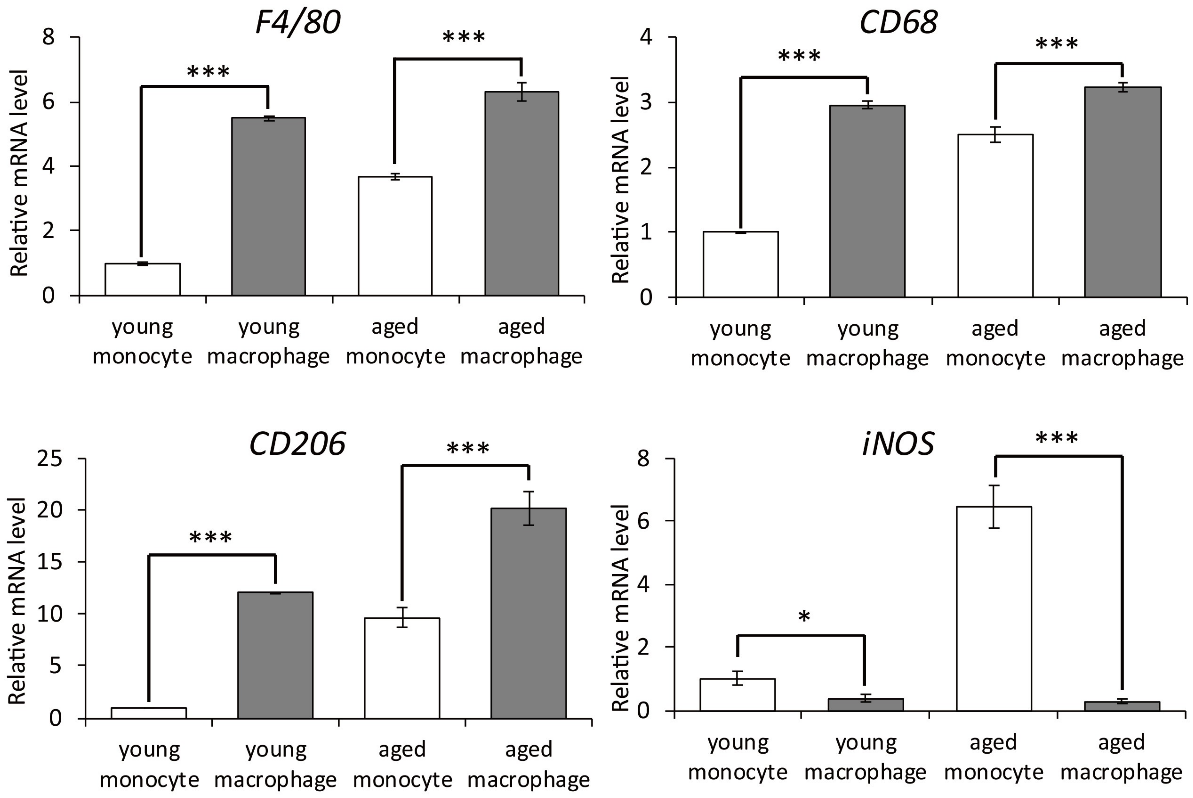

Figure S1. qPCR analysis of pan-macrophage marker (F4/80, CD68), M2 macrophage marker (CD206) and M1 macrophage marker (iNOS) in primary bone-marrow derived monocytes isolated from young (5-week-old) or old mice (72-week-old) and macrophages differentiated by RENCA cell conditioned media. Error = SD, * = p-value < 0.05, *** = p-value < 0.001 compared to monocytes.

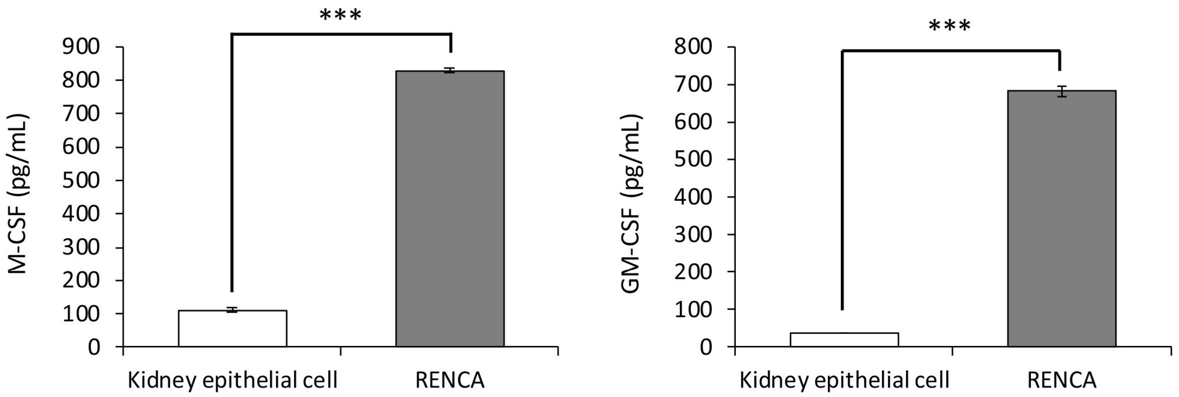

Figure S2. Quantification of factors associated with macrophage differentiation (M-CSF, GM-CSF) by ELISA in conditioned media from kidney epithelial cells and RENCA cells. Error = SD, *** = p-value < 0.001 compared to kidney epithelial cell.
